# Chemical Consequences of XUV/X-ray Laser-Matter Interactions

**DOI:** 10.3390/molecules26226833

**Published:** 2021-11-12

**Authors:** Libor Juha

**Affiliations:** Department of Radiation and Chemical Physics and the PALS (Prague Asterix Laser System) Research Center, Institute of Physics of the Czech Academy of Sciences, Na Slovance 2, 182 21 Prague, Czech Republic; juha@fzu.cz

The first soft X-ray laser was put into operation in Livermore (CA, USA) more than three decades ago [1]. Invention and implementation of pre-pulse techniques reduced a pumping power density required for the short-wavelength laser operation [2]. However, even the concept of compact capillary-discharge XUV lasers [3,4] brought the short-wavelength lasers into a standard laboratory environment. A new impulse for advanced interaction studies came with an advent of short-wavelength free-electron lasers [5,6,7]. The comprehensive information on XUV/X-ray free-electron lasers being currently in operation was summarized in Tab. 1 on page 7 of ref. [8]. Further advancement is in the field considered to be associated with a prospective successful development of compact XUV/X-ray free-electron lasers, see for example the most recent contribution described in ref. [9]. The compact FEL sources would bring the technology, now operated only at large-scale facilities, closer to the standard laboratory conditions. The chemically oriented applications should benefit from that.

Although even early considerations on XUV/X-ray laser applications taken into account an action of short-wavelength laser beams on (bio)molecular systems (see for example the cover-shown below-of the *La Recherche* (Figure 1) issue containing a review article [10] written by Pierre Jaeglé, the French pioneer in XUV/X-ray laser research) the radiolysis of macromolecular samples initiated by XUV laser beam was experimentally first observed at DESY in Hamburg two decades ago [11]. Since that time, numerous studies were published dealing with a chemical change induced by XUV/X-ray lasers of various kinds (i.e., the XUV/X-ray lasing may occur in electrical discharges, laser-produced plasmas and bunches of relativistic electrons) in molecular gases (including molecular and cluster beams), liquids and solids. 

The irradiation experiments exhibit not only scientific importance but some prospective practical applications arise from them. Although the current production technology of integrated circuits is dominated by Extreme Ultraviolet Lithography (EUVL) which does not require a high peak power of EUV sources, the XUV/X-ray lasers are capable to nano-pattern the surface directly, liberating volatile fragments of an irradiated material into vacuum (for more details see ref. [12] and references cited therein).

The other way of using the XUV/X-ray lasers to explore molecular transformations, especially their dynamics, lies in various pump-and-probe experiments where short- and/or ultra-short XUV/X-ray laser pulse acts as a probe. 

The most recent results (both theoretical and experimental ones) as well as brief reviews of earlier studies will be presented in this special issue. Not only results obtained with XUV/X-ray lasers but also experiments performed with other sources of intense XUV/X-ray radiation being able to mimic some particular features XUV/X-ray laser beams will be reported. 

## Figures and Tables

**Figure 1:**
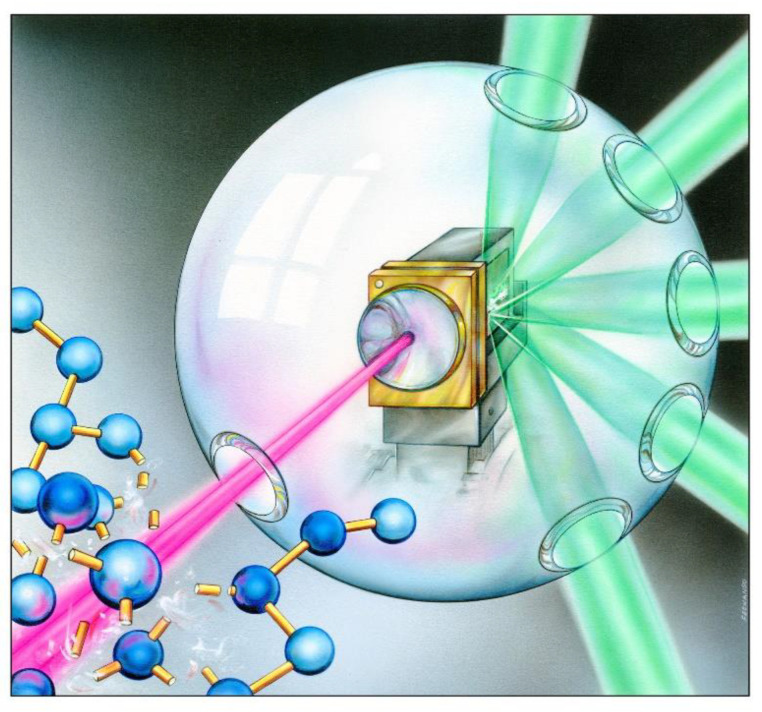
Cover of the January 1987 issue of the famous French Journal *La Recherche* showing an artist’s view on how the laser-produced plasma-based soft-X-ray laser beam (false color: violet) decomposes a molecule [10]. The artist/graphic designer: © Fernando da Cunha (reproduced with artist’s permission).

## References

[1] Matthews D.L., Hagelstein P.L., Rosen M.D., Eckart M.J., Ceglio N.M., Hazi A.U., Medecki H., MacGowan B.J., Trebes J.E., Whitten B.L. (1985). Demonstration of a soft X-ray amplifier. Phys. Rev. Lett..

[2] Nilsen J., MacGowan B.J., Da Silva L.B., Moreno J.C. (1993). Prepulse technique for producing low-*Z* Ne-like X-ray lasers. Phys. Rev. A..

[3] Rocca J.J., Shlyaptsev V., Tomasel F.G., Cortázar O.D., Hartshorn D., Chilla J.L.A. (1994). Demonstration of a discharge pumped table-top soft-X-ray laser. Phys. Rev. Lett..

[4] Benware B.R., Macchietto C.D., Moreno C.H., Rocca J.J. (1998). Demonstration of a high average power tabletop soft X-ray laser. Phys. Rev. Lett..

[5] Ayvazyan V., Baboi N., Bohnet I., Brinkmann R., Castellano M., Castro P., Catani L., Choroba S., Cianchi A., Dohlus M. (2002). Generation of GW radiation pulses from a VUV free-electron laser operating in the femtosecond regime. Phys. Rev. Lett..

[6] Ackerman W., Asova G., Ayvazyan V., Azima A., Baboi N., Bähr J., Balandin V., Beutner B., Brandt A., Bolzmann A. (2007). Operation of a free-electron laser from the extreme ultraviolet to the water window. Nat. Phot..

[7] Emma P., Akre R., Arthur J., Bionta R., Bostedt C., Bozek J., Brachmann A., Bucksbaum P., Coffee R., Decker F.J. (2007). First lasing and operation of an angstrom-wavelength free-electron laser. Nat. Phot..

[8] Callegari C., Grum-Grzhimailo A.N., Ishikawa K.L., Prince K.C., Sansone G., Ueda K. (2021). Atomic, molecular and optical physics applications of longitudinally coherent and narrow bandwidth Free-Electron Lasers. Phys. Rep..

[9] Rosenzweig J.B., Majernik N., Robles R.R., Andonian G., Camacho O., Fukasawa A., Kogar A., Lawler G., Miao J., Musumeci P. (2020). An ultra-compact X-ray free-electron laser. New. J. Phys..

[10] Jaeglé P. (1985). Le laser à rayons X. La Recherche..

[11] Juha L., Krása J., Cejnarova A., Chvostova D., Vorlíček V., Krzywinski J., Sobierajski R., Andrejczuk A., Jurek M., Klinger D. (2003). Ablation of various materials with intense XUV radiation. Nucl Instrum. Meth. Phys. Res..

[12] Burian T., Chalupský J., Hájková V., Toufarová M., Vorlíček V., Hau-Riege S., Krzywinski J., Bozek J.D., Bostedt C., Graf A.T. (2020). Subthreshold erosion of an organic polymer induced by multiple shots of an X-ray free-electron laser. Phys. Rev. Appl..

